# COVID-19 surveillance in Democratic Republic of Congo, Nigeria, Senegal and Uganda: strengths, weaknesses and key Lessons

**DOI:** 10.1186/s12889-023-15708-6

**Published:** 2023-05-08

**Authors:** Olufunmilayo Ibitola Fawole, Segun Bello, Ayo Stephen Adebowale, Eniola Adetola Bamgboye, Mobolaji Modinat Salawu, Rotimi Felix Afolabi, Magbagbeola David Dairo, Alice Namale, Suzanne Kiwanuka, Fred Monje, Noel Namuhani, Steven Kabwama, Susan Kizito, Rawlance Ndejjo, Ibrahima Seck, Issakha Diallo, Mamadou Makhtar, Mbacke Leye, Youssou Ndiaye, Manel Fall, Oumar Bassoum, Mala Ali Mapatano, Marc Bosonkie, Landry Egbende, Siobhan Lazenby, William Wang, Anne Liu, Rebecca Bartlein, William Sambisa, Rhoda Wanyenze

**Affiliations:** 1 Faculty of Public Health, College of Medicine, University of Ibadan, Ibadan, Nigeria; 2 Makerere University School of Public Health, Kampala, Uganda; 3 Department of Preventive Medicine and Public Health, Université Cheikh Anta Diop de Dakar, Dakar, Senegal; 4 Kinshasa, School of Public Health, Kinshasa, Democratic Republic of Congo; 5 Gates Ventures LLC, Exemplars in Global Health, Seattle, WA USA; 6 Bill and Melinda Gates Foundation, Seattle, USA

**Keywords:** COVID-19 response, COVID-19 surveillance, Health systems, Strengths and weaknesses, Key learnings, Lesson learnt

## Abstract

**Introduction:**

As part of efforts to rapidly identify and care for individuals with COVID-19, trace and quarantine contacts, and monitor disease trends over time, most African countries implemented interventions to strengthen their existing disease surveillance systems. This research describes the strengths, weaknesses and lessons learnt from the COVID-19 surveillance strategies implemented in four African countries to inform the enhancement of surveillance systems for future epidemics on the continent.

**Methods:**

The four countries namely the Democratic Republic of Congo (DRC), Nigeria, Senegal, and Uganda, were selected based on their variability in COVID-19 response and representation of Francophone and Anglophone countries. A mixed-methods observational study was conducted including desk review and key informant interviews, to document best practices, gaps, and innovations in surveillance at the national, sub-national, health facilities, and community levels, and these learnings were synthesized across the countries.

**Results:**

Surveillance approaches across countries included - case investigation, contact tracing, community-based, laboratory-based sentinel, serological, telephone hotlines, and genomic sequencing surveillance. As the COVID-19 pandemic progressed, the health systems moved from aggressive testing and contact tracing to detect virus and triage individual contacts into quarantine and confirmed cases, isolation and clinical care. Surveillance, including case definitions, changed from contact tracing of all contacts of confirmed cases to only symptomatic contacts and travelers. All countries reported inadequate staffing, staff capacity gaps and lack of full integration of data sources. All four countries under study improved data management and surveillance capacity by training health workers and increasing resources for laboratories, but the disease burden was under-detected. Decentralizing surveillance to enable swifter implementation of targeted public health measures at the subnational level was a challenge. There were also gaps in genomic and postmortem surveillance including community level sero-prevalence studies, as well as digital technologies to provide more timely and accurate surveillance data.

**Conclusion:**

All the four countries demonstrated a prompt public health surveillance response and adopted similar approaches to surveillance with some adaptations as the pandemic progresses. There is need for investments to enhance surveillance approaches and systems including decentralizing surveillance to the subnational and community levels, strengthening capabilities for genomic surveillance and use of digital technologies, among others. Investing in health worker capacity, ensuring data quality and availability and improving ability to transmit surveillance data between and across multiple levels of the health care system is also critical. Countries need to take immediate action in strengthening their surveillance systems to better prepare for the next major disease outbreak and pandemic.

**Supplementary Information:**

The online version contains supplementary material available at 10.1186/s12889-023-15708-6.

## Introduction

The Coronavirus disease of 2019 (COVID-19) was first reported in Wuhan city of the Hubei Province in China around late December 2019 [1]. On 30th of January 2020, the World Health Organisation (WHO) declared COVID-19 a Public Health Emergency of International Concern [2]. COVID-19 was declared a pandemic on 11th of March 2020 and by the 23rd of February, 2023, it had caused over 757 million confirmed cases and over 6.8 million deaths globally [3].

Nigeria was one of the first countries in Africa to report a case of COVID-19 on 27th of February, 2020 [4]. A few days later, on 2nd of March, Senegal reported its first case in a traveler arriving from France [5]. Uganda and the Democratic Republic of Congo (DRC) also reported cases in March 2020 [6]. Like many other African countries, these four countries experienced a relatively mild first wave of the pandemic (through July 2020) reporting a total of 17998, 35511, 87564, and 19140 for DRC, Uganda, Nigeria and Senegal, respectively [7]. The period for subsequent waves of infection differed slightly between countries. A second wave of cases and deaths followed between November 2020 and February 2021, except in the DRC where the reported burden remained low. In early June 2021, Senegal had the most reported cases and death per capita. By the middle of June through July 2021, Uganda had already overtaken Senegal and peaked in a third wave while Senegal was already experiencing a trough.

Surveillance is one of the most critical features of disease outbreak detection and pandemic response [8, 9]. The objectives of COVID-19 surveillance are to: enable the rapid detection, isolation, and management of suspected and probable cases including detecting and containing the clusters and outbreaks, especially among vulnerable populations [10]. Furthermore, surveillance is key to identifying, following up with, and quarantining contacts of confirmed COVID-19 cases at the beginning of the pandemic and providing requisite data to guide the implementation and adjustment of targeted control measures [10].

Compared with the rest of the world, COVID-19 has not been as severe in Africa. Although the African continent is home to about 14 per cent of the world’s population, the region has accounted for only about 2 per cent of reported cases and deaths as at 10th November, 2020 [11]. Low case detection rates and reporting gaps exist in COVID-19 cases and deaths across the world, and these are particularly acute in sub-Saharan Africa (SSA) [12] due to weakeness of infectious disease and mortality surveillance systems. Disease reporting gaps could partially explain the relatively lower COVID-19 burden in SSA [13, 14]. Test positivity, or the proportion of COVID-19 tests that are positive, can be used as a marker of how widespread the infection is and whether sufficient testing is being done. WHO suggests that a positivity rate of less than 5 percent is one indicator that a country has the spread of COVID-19 under control [15]. During some phases of the pandemic, especially during the second wave between November 2020 and February 2021, the test-positivity rates in all four countries were greater than 10 percent, much higher than the WHO benchmark of 5 percent, [16] suggesting a substantial undetected burden of COVID-19 by the surveillance systems.

To inform the development of sustainable and resilient surveillance systems for current COVID-19 response efforts and future disease preparedness in the region and globally, this study aimed to analyse and document the surveillance strategies in response to the COVID-19 pandemic adopted by four countries in SSA namely, the DRC, Nigeria, Senegal and Uganda.

## Methodology

### Study setting

The study was conducted in the DRC, Nigeria, Senegal and Uganda. These four countries were selected for the following reasons:

1. Variation in their COVID-19 responses, in terms of the scope and intensity of non-pharmaceutical interventions and their intended outcomes [17].
2. Historical experience in managing epidemics of global concern, such as yellow fever, Ebola virus disease, and Marburg virus disease [18–20].
3. Existing partnerships between local research institutions and government departments for ease of implementation and translation of research findings to evidence-based policy and practices.
4. Mixture of Francophone (the DRC and Senegal) and Anglophone (Nigeria and Uganda) [21] countries to enhance South-to-South cross-learning.

### Study design

**Fig. 1:**
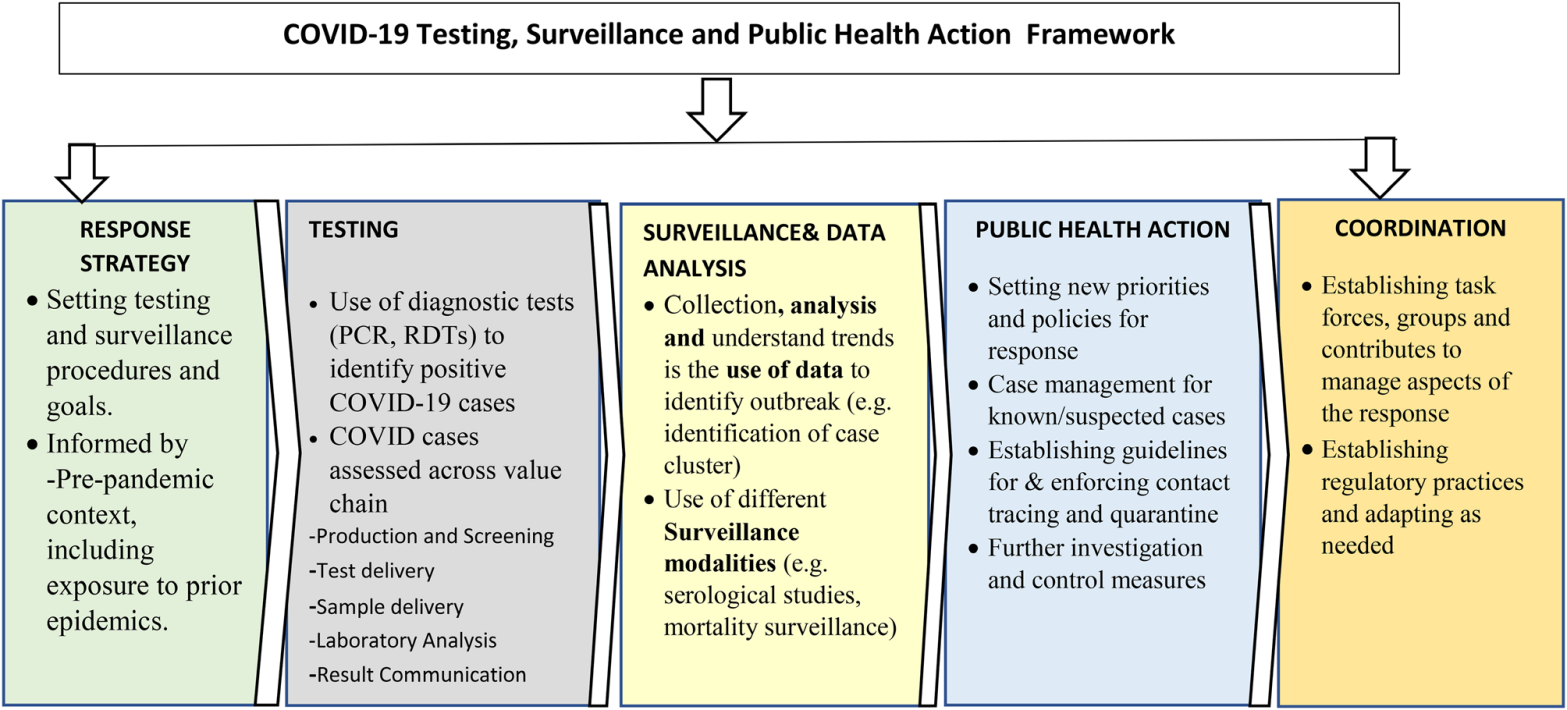
Testing, Surveillance and Public Health Action Framework Source: Exemplars in Global Health, 2021

This study used a mixed-methods observational approach based on the surveillance and public health action framework (Fig. 1). The mixed-methods consisted of; 1) a desk review of the literature and relevant documents, and 2) key informant interviews (KIIs). First, the study undertook reviews of each country’s guidelines and response plans, response reports, websites, presentations, and analysis of the COVID-19 epidemic curve. Second, researchers from the four countries conducted KIIs to further explore the issues identified in the desk reviews. Promising practices, innovations, and challenges with key recommendations for moving forward were documented and presented in this paper.

The surveillance and public health action framework has the following five (5) domains each having at least two components: Response Strategy, Testing, Surveillance and Data Analysis, Public Health Action and Coordination, (Fig. 1).First, the Response Strategy domain includes setting testing and surveillance procedures and goals, partly informed by the ‘’pre-pandemic context, including exposure to prior epidemics [21]. Second, the Testing domain has one component titled use of diagnostic tests (e.g., RT-PCR) to identify COVID-19 cases which has several sub-components namely production and sourcing, test delivery, sample collection and test demand, sample delivery, laboratory analysis, and result communication [21]. Third, the Surveillance and Data Analysis domain constitutes of the following two components, namely collection, analysis and use of data to identify and understand trends in the outbreak (e.g., identification of case clusters) and use of different surveillance modalities (e.g.,, serological surveys, mortality surveillance). Then the Public Health Action domain constitutes four components namely setting new priorities and policies for response, case management for known and suspected cases, establishing guidelines for contact tracing and quarantine, and investigation and control measures. Finally, the Coordination domain constitutes two components which are establishing task forces groups and committees to manage aspects of direct surveillance and testing activities, and establishing regulatory practices [21]. This study focuses on only the surveillance and does not explore aspects of testing which is being addressed in another publication [21]. The study also explores how other components such as response strategy, public health action and coordination influence surveillance and data analysis.

### Study population

Study participants were selected based on their role in the COVID-19 response. They typical include policy makers and members of the national and regional emergency operations centres for COVID-19 response. Informants were identified and selected from national public health institutes, Nigeria centre for disease control, state ministry of health and local/district health authority. Research assistants visited the offices offices of the informants at their convenient time to conduct the KIIs. The study conducted (KIIs) with 30 informants per country, including policy makers, epidemic focal persons, and health managers.

### Study instrument

Two data collection instruments were used (Supplementary Materials). The first instrument was a literature abstraction form (Additional file 1) used to gather information across the four countries on testing modalities, changes in testing criteria, modalities of implementing contact tracing, isolation, screening and the surveillance modalities for COVID-19. The second instrument was a KII guide (Additional file 2) which was used to obtain information on the strengths and weaknesses of the surveillance strategies in the four countries. The KII themes under which information was gathered were past experience with epidemics, health system preparedness (specifically about funding), response to the COVID-19 pandemic in Africa and the outcomes in terms of non-pharmaceutical COVID-19 control strategies, including strategies to ensure continuity of essential non-COVID services.

### Data collection

The data extraction form was developed and piloted in the excel format but was also applied in the word document format to extract data, depending on preference by country teams. The sections relevant to surveillance extracted data on strategies including; modalities of contact tracing, modality of implementing isolation, modalities of screening, and surveillance data. For each strategy, data on key strengths, weaknesses, challenges and gaps were also extracted where reported. Investigators conducted search and extracted data on different sections independently.

In each country, data collection was facilitated by skilled research assistants with proficiency in qualitative research. The research assistants received additional training on data collection strategies and use of the guide. Between February and March, 2021, 30 KII were conducted in English (Nigeria and Uganda) and French (the DRC and Senegal) among policy makers, program managers, and epidemic response and health system implementers and managers to further explore the issues identified in the desk research.

Prior to conducting the KIIs, the research team conducted a desk research. The mixed-methods observational approach was utilized to facilitate the triangulation of information to enable a wide-ranging documentation of the strengths, weaknesses, gaps and innovations in surveillance at the national, sub-national, health facilities, implementing partners and community levels. Key learnings both similar and unique to a specific country context were synthesized across the countries.

### Data management

The data from the literature review were synthesized thematically. All KIIs were audio-recorded on mobile phones and voice recorders. Codes were assigned to each key informant to ensure the confidentiality of the participants. The recorded data were transcribed, cleaned and coded. Thematic analysis was done using Excel and Atlas Ti software packages. The recordings and notebooks were securely stored in locked cabinets and password protected.

### Ethical considerations

Each country research team obtained ethical clearance from relevant national and Institutional Review Boards. Clearance was obtained as such DRC- No d’Approbation: ESP/CE/198/2020; Nigeria-NHREC/01/01/2007; Senegal-000279/MSAS/DPRS/DR 03/03/2021; and Uganda-UNCST HS 1121ES/HDREC 903. The study was conducted in accordance with the Declaration of Helsinki. The permission of the gatekeepers’ was obtained to allow access to the facilities. Before conducting interviews, the purpose of the study was explained to the participants who provided written or verbal (for respondents who desired more anonymity) informed consent depending on the country. Importantly, participants were reassured of confidentiality of their responses and anonymity.

## Results

### Literature review

All four countries are committed to the Global Health Security Agenda (GHSA), and the International Health Regulations 2005. Furthermore, they all are implementing the Integrated Disease Surveillance and Response [21] system [22]. Table 1 shows the pre-pandemic health context in the four countries. Nigeria is the most populous of the four countries (215 million) compared to Senegal’s 17 million. On the other hand, Senegal had the highest life-expectancy of 68.5 years while Nigeria has the lowest at 54.3 years. Uganda has the best universal health care coverage index of 53 compared to the + DRCs 45 [23, 24]. Prior to the COVID-19 pandemic, WHO and MOH collaborative evalutions found that all four countries had very limited capacity in the following technical area across all levels of the epidemic preparedness of the health system namely detection, response and control of public health threats (WHO JEE reports: DRC-2018; Nigeria-2017; Senegal-2016; Uganda-2017). The COVID-19 epidemic underscored the critical role of surveillance in protecting individual nations and the global community.

**Table 1:** Pre-Pandemic Health Context across the 4 Countries

Country	DRC	Nigeria	Senegal	Uganda
Population	92 million	206 million	17 million	42 million
Life expectancy from birth (Japan and Singapore have longest, about 85years)	65.1years	64.3yrs	68.5yrs	66.2yrs
Universal health care effective coverage index (0-100)	45	38	50	53
DTP3 coverage in children in 2019	48%	50%	93%	87%
In-facility births	85%	49%	84%	79%
Maternal mortality ratio (SDG3.1 is 140/100,000 by 2030)	345 per 100,000 live births	233 per 100,000 live births	379 per 100,000 live births	133 per 100,000 live births
Doctors, nurses, midwives per capita. Benchmark is 4.45 to achieve universal health coverage	1.2:1000	1.6:1000	0.4 :1000	1.4 : 1000
Total health expenditure per capita (% of GDP). Benchmark in health financing is $86/person or 5% of GDP to achieve Universal Health Coverage	$20 3.6%	$83 3.5%	$65 4.7%	$48 6.4%
Out of pocket health expenditure (% of total health expenditure)	$9 per capita 45%	$63per capita 76%	$31 per capita 47%	$19 per capita 40%

Source: Institute for Health Metrics and Evaluation. Global Burden of Disease study 2019; Institute for Health Metrics and Evaluation Financing Global Health; World Bank Data Repository

Surveillance for COVID-19 was risk-based across all four countries and the prevention, control and mitigation strategies implemented had many similarities. Initially, the priority groups for contact tracing were all contacts of confirmed cases, travelers originating from countries that had reported COVID-19 cases, and health care workers exposed to confirmed cases, but as the pandemic progressed the prioritization was revised to include only symptomatic contacts and travelers. To implement contact tracing, additional human resources were engaged either through recruitment of volunteers or contract staff. Initially, contact tracing was centrally coordinated and targeted all contacts, but as the epidemic progressed to widespread community transmission and the cases increased, the activity was decentralized and tracing targeted individuals at higher risk of severe disease. Effectiveness of contact tracing during early phases was generally good, with over 90% of contacts reportedly traced in all the countries [25].

### Morbidity and mortality due to COVID-19

Table 2 shows data from literature on the morbidity and mortality patterns due to COVID-19 in the four countries under study. As at April 2022, Senegal had the highest number of cases and deaths per million and the DRC had the least (5000 and 114.27 versus 939.05 and 14.47 per million respectively).

**Table 2:** Cumulative Morbidity and Mortality due to COVID-19 across the 4 countries

Location	Reported Cases	Reported Deaths	Population (in millions)	Cases per millions	Deaths per millions
World*	491,440,000	6,150,000	7,870	62,410	781.32
**Africa**	11,560,000	251,990	1,370	8,420	183.47
DRC	86,750	1,340	92.38	939.05	14.47
NIGERIA	255,470	3,140	211.4	1,210	14.86
Senegal	85,920	1,970	17.2	5000	114.27
Uganda	I63,940	3,600	47.12	3,480	76.31

Source: Our World in Data, Johns Hopkins University CSSE COVID-19 Data [21]

### Synthesis of literature review and key informant interviews

#### Surveillance methods and Systems

Across the study sites and contexts, a networked and combination of different surveillance methods were used to get a holistic picture of the spread of the COVID-19 disease and behavior of the populations served by ministries of health and donors. The examples of methods used across the four countries can be classified into active and passive surveillance. The active surveillance included case investigation, contact tracing, community-based surveillance, sentinel site surveillance, serological survey, event-based surveillance, telephone hotlines, genomic sequencing and pathogen surveillance and environmental surveillance. The passive surveillance included surveillance at the primary care level, laboratory-based surveillance, hospital/facility-based surveillance, point of entry surveillance, work-based surveillance, mortality and postmortem surveillance. These surveillance systems have been linked to electronic data collection and reporting systems e.g. District Health Information Sysytem (DHIS-2) and Surveillance Outnreak Response Managaement and Analysis System (SORMAS) for real time access. Findings from the KIIs also buttressed the adoption and use of these various methods. Active case findings were adopted in community based surveillance across countries such as Nigeria, Uganda and DRC. Community health workers and village health teams collaborated with community members in some instances for community ownership of the tracing process. One key informant noted that:

*“Community health workers conducted community surveillance through active case searches as well as the national COVID-19 hotline, which managed nearly 3,000 calls per day by November 2020. Community health workers also were involved in contact tracing and supported outreach in each health zone” (****MoH, Epidemiological Surveillance Directorate, DRC)***

Networks of laboratories coordinated and funded by the government and partners such as the CDC Atlanta, were set up and connected across regions of the countries for laboratory-based surveillance. These laboratories as well as facilities involved in facility-based surveillance were linked to SORMAS for reporting as mentioned by a respondent:

*“We set up laboratories almost all over the state in Nigeria and this all pulled down to the hub, the national reference laboratory situated at Gaduwa in Abuja. And this also helped to ensure testing and ensure that the laboratory-based surveillance is been done for COVID-19”****(Case Management Pillar Member NC, Nigeria)***

“Facility based surveillance *was done to obtain information from people in the hospitals. The data was included into SORMAS, and that was where we got some information for trend, transmission peculiar to Nigeria unlike the other parts of the world, assessment, demographic figures concerning COVID-19. We discovered that COVID cut across all ages, even newborns tested positive. It is also the same risk for men and women, but more in men. All these information peculiars to our environment were derived from our own data”* ***(Laboratory Team Lead, State Emergency Operations Centre [EOC], SW Nigeria.)***

Across countries under review, air, land and maritime borders were subjected to point of entry surveillance. Trained personnel were deployed to screen travelers and contacts of suspected cases. A key informant from Senegal described this:

*“surveillance has always been daily but it has been reinforced with the arrival of this pandemic... surveillance has been reinforced, particularly at the border level because there is a flow of travelers which means that the risks were as much as possible, particularly at the level of air borders, but we have also not forgotten the surveillance of maritime and land borders...“* ***(Technical Manager at MoHSA-Senegal)***

Furthermore, work-based surveillance was instituted in most work places with preventive instructions such as staff being instructed to work from or stay at home when ill as described by a key informant:

*“Well... work-based surveillance was basically introduced through Infection Prevention and Control (IPC)* [26]. *So, IPC people were trained on how to identify these cases. First of all, surveillance is basically about identifying a case and reporting. That’s surveillance. Seeing and knowing institutions that this it is. So in offices, people who were seen to have had respiratory illnesses were asked to stay at home, get tested and come back to work when they are well. So, that was the form of surveillance that was done* ***(Surveillance Officer, Uganda)***

Mortality and postmortem COVID-19 surveillance was implemented to prevent infection to bereaved families during preparation for burial for the deceased because of delayed test results as indicated by a key informant:

*“We had some cases that their sample were taken for investigation. Some people tested positive after death. Other things might have killed the patients, but we were able to confirm COVID-19”* ***(Laboratory Team Lead, State Emergency Operation Centre (EOC), SW Nigeria)***

**Fig. 2:**
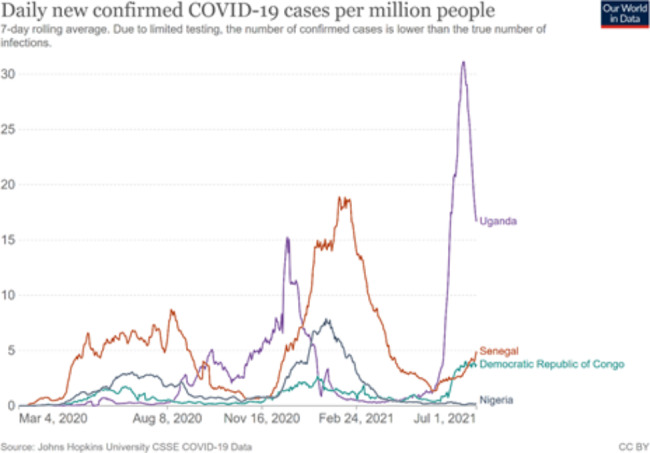
Daily new confirmed COVID_19 cases per million

**Fig. 3:**
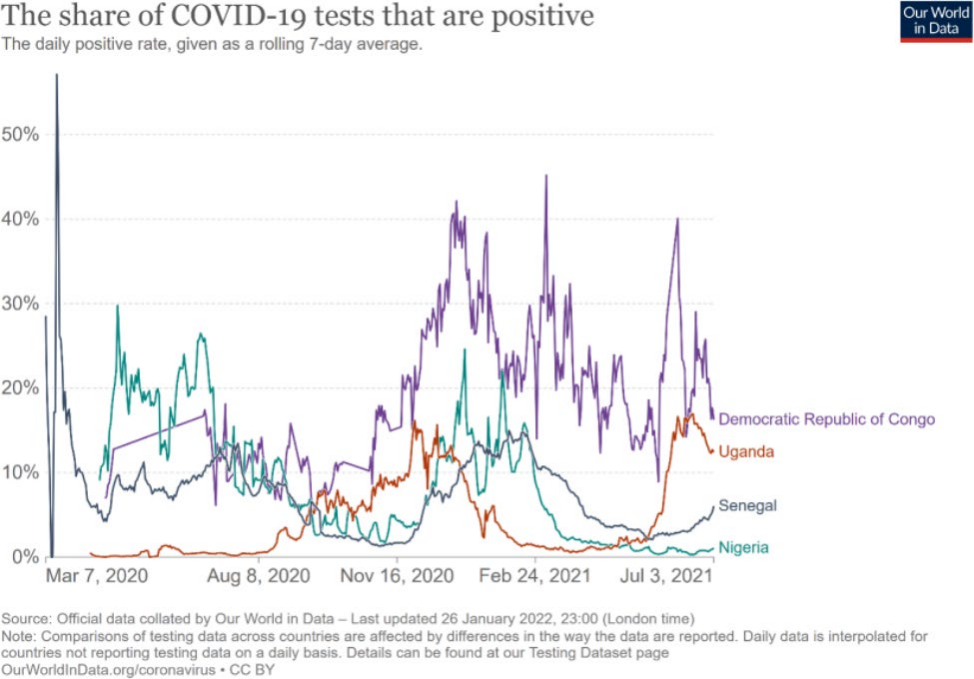
The share of COVID_19 test that are positive

**Sentinel site surveillance.** Population-based surveys of antibody sero-positivity and the use of serology in specific settings or populations to estimate the proportion of the population that had been infected with SARS-CoV-2 were done by all. Uganda conducted three community surveys and testing for COVID-19, twice in 2020 and once in March 2021, to determine the community burden to complement the surveillance efforts. Also, Nigeria conducted a national serological survey (September-October 2020 that found up to 23% in Lagos had COVID-19 antibodies, much higher than expected [12]. Later, another survey was conducted in May-June 2021 with report which is not yet in public domain. In June 2020, the DRC conducted ‘mass’ COVID-19 testing in one section of the capital city, Kinshasa, the epicenter of COVID-19. On the other hand, Senegal conducted a national survey in October, 2020. Figures 2 and 3 show the daily new confirmed COVID-19 cases per million people and the proportion of COVID-19 tests that are positive, respectively.

#### Key strengths of the surveillance approaches employed

Table 3 below describes key strengths and challenges of the surveillance system that were observed in the study countries.

**Table 3:** Unique Strengths in Surveillance and Data Management across the 4 countries

	DRC	Nigeria	Senegal	Uganda
**Surveillance**	On alert due to ongoing EVD epidemic: leveraging EVD preparedness for COVID-19 Existence of a well-organized and trained surveillance team at all level of the health system (including CHWs) Well established functional laboratory networks that allowed for rapid ramp up of testing capacity Experience in dealing with different outbreaks Conducted mass testing in one region of Kinshasa	Leveraged pre-existing SORMAS software and DHIS2v EWARS functional in conflict areas Engaged private labs in tracking travelers for post quarantine for COVID-19 testing Community sero-prevalence completed in 6 states (2020/2021) Strong central coordination to avoid duplication and proactive response before first case was recorded Strong financial support from federal government	Establishment of a national alert set up during the preparatory phase with a dedicated short number accessible 24/7 for case detection. Analysis of the distribution of confirmed cases was done to identify 45 high priority districts that guided response	On alert due to ongoing EVD epidemic: leveraging EVD preparedness for COVID-19 Restructuring of surveillance pillar i.e. creation of sub pillars including; health worker surveillance, alerts, quarantine and Points of Entry teams Community surveys – Two Rapid Assessments for COVID-19 prevalence conducted April and August 2020. The surveys used RT-PCR (for active infection). A third was conducted March 2021 Pre-existing functional national laboratory network Scientific advisory committee fed the NTF helping with rapid translation of data, emerging issues, research priorities, and policy shifts. The availability of a research and innovation fund [35] from government paved the way for innovations
**Data management**	Introduced e-surveillance at all levels of health system August 2020 for enhanced data collection and reporting EWARS is used in some pilot health zones for management of alerts	Real time surveillance dashboard Automated epidemiological bulletins generated to rapidly analyze and share results EWARS used for line listing and management of data Testing labs linked to DHIS2	Establishment of software (Daan COVID) facilitated data collection and analysis for clinicians and surveillance system Introduced MoH COVID 19 tracker module which is able to cover all needs for information	Innovation of electronic tools and systems including the interactive voice response systems, ODK, HMIS, RECDTS, eIDSR, DHIS2, Go Data for case reporting, detection, investigation and follow-up An electronic integrated Disease Surveillance Response (eIDSR) was integrated into DHIS2 to capture real-time data and monitoring Centralized data bases with an electronic Results Dispatch System (eRDS), with downloadable electronic results improved reporting of lab results Integration of lab data into surveillance reports

1. **Leveraging pre-existing surveillance systems for COVID-19 surveillance.** All four study countries had existing functional surveillance systems networked with national laboratories as well as have trained and experienced surveillance teams with in-country epidemiologists capacities [27, 28]. The previous disease outbreak experiences contributed to rapid preparedness planning for COVID-19 and use of the existing surveillance capacities and capabilities. For example, during the oubreak of COVID-19, the DRC had an ongoing Ebola epidemic and had trained and mobilized surveillance officers while Uganda’s community surveillance officers had been recently trained in preparation of possible cross-border transmission of the DRC Ebola outbreak (2018–2020). These active and preparatory capacities for the Ebola outbreak were immediately available to support COVID-19 surveillance. Previous experiences with similar outbreaks helped the two countries to rapidly ramp up surveillance capacity [12, 29]. Similarly, Senegal and Nigeria had experienced previous Ebola disease outbreaks and established relevant surveillance systems.

*“For the experience we have gained in the management of previous epidemics, it is important to know that Senegal is not at its first epidemic, we have had to manage a lot of epidemics…take as an example the Ebola virus disease even if we had only one case it is this epidemic experience allowed us to strengthen our system and it is precisely after this epidemic that the COUS Health Emergency Operations Center was created with the main mission of preparing the response to the public health emergency” –****(Member of the*Emergency Operation Center**, ***Senegal)***

2. **Capacity enhancement**.All the four participating countries strengthened surveillance capacity at sub-national level by training rapid response teams at district/regional/provincial/state levels, with support from development partners [30]. Sub-national teams were pivotal in supporting lower-level structures including community health workers to conduct contact tracing. Training, dissemination of policies and tools (i.e., data collection forms, case definitions, and reporting tools) to support surveillance occurred in a timely manner. However, all four countries reported staffing shortages especially for dedicated surveillance officers at the local levels of the operation of the surveillance systems.

*‘’Not only Village Health Teams (VHTs) but we also brought the community leaders on board to do surveillance, and that is working for us very well…the community is leading the surveillance rather than for us* [31] *owning the activity”(****District Surveillance Focal Person, Uganda****)*

*“In Uganda the use of community health workers to implement COVID-19 surveillance was a strength, while in DRC, the use of Early Warning, Alert and Response System (EWARS) (Early Warning, Alert and Response System) was a strength”* ***(Surveillance officer, DRC*** *)*

3. **Data management reporting and use**: All the four countries had established databases with capacity to report on key indicators on the status of the response to COVID-19. These countries built the reporting of COVID-19 indicators on existing data management systems for collecting, collating, analysis and reporting on the performance of these indicators. All countries adopted electronic systems to improve timeliness of reporting. The reporting systems were linked to the current (DHIS-2) system which enhanced storage, retrieval, analysis, and presentation as well as interpolation with existing data. Data was shared through dashboards [32], bulletins, situation reports and was accessible to key decision makers to support the response. The availability of scientifically sound data contributed to the regular review and update of surveillance policies and strategies. The use of digital platforms, short text messages and telephone calls was reported by all countries for sharing results with decision-makers and for improving the health of the population in their jurisdiction. However, interoperability and data quality were still key challenges.

*“To the best of my knowledge I will say [SORMAS] is near perfect…because we are not using the outdated paper-based surveillance system, we are using the e-format…which is like ‘real time’, because as soon as the result is received from the lab, the data manager inputs the results electronically, and at that point everybody who is a stakeholder sees it and can respond” (* ***Capacity Building Trainer, NC, Nigeria*** *).*

*“In June 2020, with technical assistance from the health systems firm Bluesquare, some health zones piloted digital reporting to replace the paper-based reporting in use under the Integrated Disease Surveillance and Response strategy. The objective was to incorporate COVID-19 indicators into DHIS2 and improve the timeliness of case reporting. In addition, WHO started piloting the Early Warning, Alert and Response System (EWARS) in some health zones. However, as of December 2020, the Bluesquare and WHO systems were not fully integrated” (* ***MoH, Epidemiological Surveillance Directorate, DRC*** *)*

“*The tracker is a module that has been developed, and which makes it possible to follow all the cases as well as their contacts” (***DHIS manager-Senegal)**

4. Multisectoral engagement and partnerships: Each country established or leveraged robust partnerships with non-governmental organizations, academics, and other global institutions.

##### Key challenges and gaps

The following challenges with the surveillance systems were observed in the four study countries [26]:-.

1. **Inadequate human resources -** The four countries lacked adequate human resources to trace contacts, in addition to testing and insufficient space for institutional quarantine and isolation for individuals tested as COVID-19 positive. For example, in Uganda the shortage of surveillance officers at the facilities affected contact tracing and other public health actions. The lack of appropriately trained human resource was more prominent at the lower levels. Similar challenges were reported in the DRC, Senegal and Nigeria.
*“Yea, some of the gaps are in terms of the level of human resources, because whether we like it or not even though the response is being coordinated at the National level it still needs to get them to the sub-national level. So, there are issues of inadequate human resources and also financial resources too. Response required a lot of resources that were not available”* ***(Case Management Pillar Member, National EOC, FCT, Nigeria)***
2. **Low case detection rates -** In the later phases of the pandemic, COVID-19 case detection was suboptimal because of limited capacity in terms of testing and workforce, and as a result of the shift to the risk-based testing strategy that the four countries adopted. The serological survey conducted in Nigeria confirmed that infection rates were over 20% and much higher than reported. In Uganda, the reported ‘probable deaths’ were comparable to the confirmed deaths which suggested under testing and underreporting of cases and deaths. Furthermore, there was underreporting of cases in both the DRC and Uganda.
*“The current epidemiological situation is only the tip of the iceberg. A large part of the cases is not detected” (* ***Member, Presidential Committee for Epidemiological Surveillance, DRC*** *).*
  *“In the early part of this outbreak, everyone was reporting COVID-19 and it was difficult and expensive for us to do verification therefore, we missed cases”* (**Regional surveillance focal person, Uganda**)
**“** *Many people were hiding and did not want to admit they had COVID because they did not believe that the existed, this meant that cases that were probably and died in the community without being detected. Thus, a large number of deaths in community escaped the vigilance of epidemiological surveillance* ***” (Member, National Committee for Epidemiological Surveillance, DRC).***
3. **Limited genetic sequencing -** The surge in cases through December 2020 was reported to be due to coronavirus variants with increased transmissibility [33]. However, the four countries had limited capacities for routine genotype tracking of the transmission dynamics and for planning. For example, in the identification of effective vaccines, it is important to know the circulating virus strains. Senegal and Nigeria had both reported presence of Omicron (November/December, 2021) and Delta (July 2021) mutant variants in the population while DRC identified omicron variant in December 2021. The omicron variant coincided with the resurgence of seasonal influenza, which has some symptoms similar to those of COVID-19, including prolonged cough, fever, headache, fatigue, body aches, weakness while Uganda identified first case of omicron by December, 2021 [34].
4. **Decentralized surveillance capacity was generally weak -** In Uganda, all the districts could not implement surveillance activities without support from the national level. Nigeria reported the State EOCs were weak and not routinely using the available data.
*“For COVID-19 surveillance to be improved, part of what I think the country can do more is that to make sure that interface between SORMAS and other system that are being use by parastatals are made seamless and to encourage more states to key to the SORMAS system, to make sure that there are more trainings, which they have being doing, with key stakeholders, surveillance leads or surveillance department team in every state of the Federation* **”** ***(Case Manager, State EOC, NC, Nigeria)***
*“Uganda experienced challenges with response at the EOC and were overwhelmed with the COVID-19 cases. “We lost time until a point of inefficiency when many calls overwhelmed the EOC call center where some were not being picked or attended to, until we decentralized” (* ***Surveillance Officer, Uganda*** *)*
5. **Insufficient space for quarantine and isolation -** In all four countries, there was insufficient accommodation space for quarantine and isolation. For example, at the beginning of the COVID-19 response in Nigeria, quarantine and isolation was carried out in government approved hotels, and in some instances all confirmed cases where hospitalized irrespective of disease severity. Initially the governments paid for hotel accommodation and general upkeep, however, later incoming travelers were required to pay their hotel fees. These deficiencies made contact tracing and surveillance more challenging, especially with increasing case volume. Across all study countries, as the pandemic gained momentum and funds became depleted, the institutional quarantine and isolation strategies were changed to home based quarantine and isolation. Compliance with self-isolation and self-quarantine became a challenge in the drive to prevent and control the epidemic resulting in increased community transmission of the virus [35].
6. **Weak surveillance infrastructure -** The health infrastructure in all countries was weak and not adequately situated to manage a global pandemic. There was inadequate health manpower and funds designated for COVID-19 surveillance (Table 4). Surveillance tools and logistics were also not readily available in the early stages of the pandemic. The decentralized and community surveillance structures had varying degrees of performance and required additional support especially in data analysis and use, postmortem surveillance and tracking of excess deaths across the study countries.

**Table 4:** Unique Challenges and Key Learnings in Surveillance and Data Management across the 4 countries

	DRC	Nigeria	Senegal	Uganda
**Surveillance**	Expansion of the e-surveillance Testing of all close contacts of a confirmed case (even when asymptomatic) limited by limited test kits Multiple concurrent epidemics (Ebola and Measles) shifting attention and stretching resources Inadequate funding and resources to manage multiple outbreaks Connectivity challenges affecting roll out of electronic systems, system remained mainly paper based. Multiple reporting systems	Hard to reach areas Conflict areas of the North East Logistics for adequate contact tracing Big geography, inadequate laboratory support in parts of the country	All community cases are not documented and there is delay referring to health facilities for some community cases. Limited engagement of scientific team on the national task force delayed interpretation / translation of emerging data and findings into policy with e.g. delay in conducting serological surveys	Delay in evacuation of some positive cases Limitations in timely case detection, investigation and reporting at the district level Centralized EOC with limited use of data by the subnational structures.
**Data management**	Existence of multiple data systems renders the integration and use of data very difficult Not all health zones have acquired the digital system Poor internet connectivity in some areas and lack of Tablets and air time	Risk of suboptimal reporting due to stigma Poor use of data to guide decision making at subnational level and some states faced coordination issues	Non-systematic analysis and discussion of data collected and regularly disseminated Community stigma and misinformation influenced demand Multiple reporting applications and mechanisms made coordination difficult Under-detection of cases and variants	Dwindling interest in active reporting from districts Overreliance on donor funding and foreign supplies Limited resources for district coordination
**Key Learnings**	Task-shifting to community health workers for contact tracing Develop hotline for case reporting	Leverage experience & systems from past outbreaks Adopt tech solutions that integrate disparate information systems	Leverage government leadership for national communication strategies Enhance multi-sectoral partnerships to boost capacity and innovation	Rapid response and proactive action Initiate community surveys Leverage available funding for innovation

*“When community transmission took off and we had overwhelming cases, the contacts were also too many yet the resources for the district teams to move to where cases had been identified, contact listing and monitoring were limited and it became impracticable to follow up contacts so that arm of surveillance contact tracing to detect cases has over time become very limited in its implementation” (* ***Surveillance Officer, Uganda*** *).*

7. **Community stigma and misinformation** - There was poor public perception of the cause and prevention of COVID-19, and there were many misinformation and conspiracy theories about the pandemic on social media across all the four countries. This affected the care seeking behaviors of the populations including testing among contacts and symptomatic individuals and ultimately the disease surveillance.

*“Another challenge was the perception of the public… there was a lot of misinformation… We had several negative experiences with contacts and their families. Logistics was also an issue as we didn’t have enough vehicles to do contact tracing. Some of us were working with our personal cars and we wouldn’t get reimbursed when we hired vehicles. It was quite daunting as we didn’t have much technical manpower. In one day, I trained 3 sets of contact tracers”* ***(Surveillance Pillar Member, State EOC, SS Nigeria)***

*“People are not ready to cooperate with us most of the time because they are afraid and ashamed, it affects surveillance because the confirmed case will have exceeded the incubation period before we can get to them”* ***(Laboratory Team Member, State EOC, SW, Nigeria)***

*“The problem of surveillance is the non-disclosure of suspected cases, the population considers this as a denunciation / betrayal vis-à-vis their parents or their neighbors. This shows how the disease is perceived by populations, it is a shameful disease.* ***(Member of Health District Team Management-Senegal)***

**“** *It was not easy to manage COVID-19 because in all the structures, people did not want to believe that the disease existed. For most Congolese, COVID-19 is an invention of the whites to eliminate the Africans* **”** ***(Member, National Committee for Epidemiological Surveillance, DRC).***

## Discussion

### Summary of findings

Disease surveillance systems are developed for the monitoring of the health status of populations and most importantly for early detection of infectious diseases outbreaks and prompt intervention. Surveillance is a top priority in management and control of any pandemic. Countries’ responses to emergent health crises depend mainly on the strength of the surveillance systems they establish [36]. This study aimed to document the COVID-19 surveillance strategies adopted by DRC, Nigeria, Senegal and Uganda in response to the COVID-19 pandemic as well as to describe the strengths, weaknesses, and lessons learnt about the existing surveillance approaches adopted during the epidemic. Furthermore, the aim of the study was to gather evidence on functionality of the adopted surveillance so as to inform the enhancement of surveillance systems to facilitate preparedness for future epidemics in Africa.

Surveillance for COVID-19 cases was risk-based across all four countries and involved a combination and networking of several surveillance methods. It was found that all countries had previous experiences with managing surveillance systems in an epidemic situation such as Ebola disease and were therefore, able to respond promptly to the pandemic but insufficiently due to resource constraints. For example, the national Emergency Operation Centre (EOC) in Nigeria had been in existence and had ample experiences in polio as well as in Ebola outbreak surveillance and response. The key strengths across the studied countries included leveraging on these previous outbreak experiences and pre-existing functional surveillance systems, strengthened surveillance capacity at sub-national levels by training rapid response teams at subnational levels; establishment of databases with capacity to report on key indicators on COVID-19 response, including electronic systems linked to DHIS-2 which contributed to regular review and update of surveillance policies and strategies. Developed countries on the other hand have standardized surveillance response system with skilled technical staff.

All four countries adopted realtime and active contact tracing as one of the essential surveillance approaches so as to control the disease through empowering decision makers with information on the health-related behaviors of their communities and the spread of the COVID-19 disease in the community. This enables the governments of the study countries to intervene quickly to stop the spread of disease. The priority groups for contact tracing included high-risk persons such as contacts of confirmed cases, and travelers from countries with reported COVID-19 cases, but prioritization was revised to include only symptomatic contacts and travelers, as the pandemic progressed. Furthermore, contact tracing was decentralized at a later time to target individuals at higher risk of severe disease.

The key challenges and gaps included; inadequate human resources for surveillance activities especially at lower levels; insufficient space for institutional quarantine and isolation; low case detection rates; limited capacity for routine genomic sequencing of variants; weak decentralized surveillance capacity; insufficient infrastructural capacity for quarantine and isolation; weak health care infrastructure including inadequate funds and tools for surveillance activities; misinformation and poor public perception about COVID-19, especially on social media. For example, inadequate human resources limited the optimal performance of surveillance systems. This was corroborated by the overwhelming load of contact tracing workload for healthcare workers reported in other studies where Uganda had 186/100,000 population and Nigeria had 111 per each state with populations in millions [25]. There was also humanitarian assistance from donors such as WHO, UN baskets, non-govermental organizations and the Nigerian indigenous Coalition against COVID-19. The world bank provided funds for contact tracing and Africa Centres for Disease Control and Prevention funded active case search.

Inadequate human resources and other challenges moderated the gains that would have accrued from adapting already existing surveillance systems and experience in managing outbreaks. The UK support initiative and Clinton health access initiative supported state and local capacity building and ongoing vaccine development.

As the pandemic progressed, the resources available could not cope with upsurge in activities required to maintain standard operating procedures leading to modifications in surveillance strategies. The criteria for testing and contact tracing were streamlined to reduce the workload. Isolation and quarantine facilities were expanded to private facilities with the implication of poorer follow-up and monitoring. Furthermore, all countries reported gaps in data management and surveillance response at subnational levels. The importance of task shifting to community health workers, adopting technology based solutions, strong national leadership including enhancing multisectoral partnership to respond to the pandemic was adopted. The countries prioritized national-level coordination of the various surveillance approaches across sectors and stakeholders. Each country established or leveraged robust partnerships with non-governmental organizations, academics, and other global institutions. These countries improved data management and surveillance capacity rapidly by training health workers and increasing resources for laboratories, but the disease burden continued to be under-detected.

### Results in context of the literature

A key objective of the World Health Organisation’s COVID-19 surveillance is to guide the implementation and adjustments of COVID-19 control measures including isolation of cases, contact tracing and quarantine of contacts [37]. The experience of African countries in handling previous infectious disease outbreaks and the existing surveillance infrastructure were helpful and in part made Africa fare better in COVID-19 pandemic compared to the high-income countries [38]. The exising surveillance infrastructure was revitalized and repurposed for COVID-19 surveillance. The surveillance methods documented in this study was corroborated by a systematic review of COVID-19 surveillance systems in 13 other African countries which documented the similar surveillance methods reported in this study [21]. Some variations however, exist in the level of implementation of the surveillance strategies between the countries which determines to a large extent, the representativeness of the systems. South Africa with more comprehensive surveillance system reported more representative COVID-19 burden data compared to countries such as Tanzania and our four study countries where COVID-19 surveillance strategies were poorly developed [21]. The interpretation of the morbidity and mortality burden from COVID-19 is subject to the quality of the surveillance system adopted. The surveillance systems in the four African countries under study have been generally non-representative of the entire underlying population. The seroprevalence surveys conducted in these countries reported a much higher COVID-19 prevalence than would be expected by the number of cases reported.

Therefore, there is a need for these countries to utilize multiple surveillance approaches to understand the full picture of the disease burden. The risk based testing strategy adopted by the countries underestimated the burden of COVID-19 due to underreporting. Extending COVID-19 testing to all contacts of a confirmed case would give a better sense of the disease burden but this attracts high cost which the countries may not have the capacity to afford and sustain. Hence, additional sources of data may be needed for example from mortality surveillance, and community surveys which were not fully integrated. A study in Zambia found significant excess mortality due to COVID-19, with the majority of deaths occurring in the community that were undiagnosed, while many deaths at the facility were also un-tested prior to death [39]. Mortality surveillance and all-cause mortality tracking has generally not been widely practiced in many African countries. Uganda attempted to establish a mortality surveillance system and has been conducting post-mortem surveillance for hospital and community COVID-19 deaths, but this has remained weak due to challenges in mortality reporting in the health systems. In Nigeria, the serological surveys conducted confirmed much higher COVID-19 prevalence than reported through the regular surveillance system, thereby emphasizing the importance of using multiple surveillance methods.

### Implications for epidemic preparedness and response

During the epidemic these countries made efforts to upgrade and upscale the health systems infrastructure so as to improve resilience and to enhance rapid response to infectious diseases emergencies.

Generally, all the four countries started planning early and had a fairly slow buildup of COVID-19 cases. The study revealed the existence of a emerging framework for surveillance structure and system in the four countries. At the onset of the pandemic, all four countries under study had in place limited existing surge capacities mainly in the areas of laboratory testing and trained epidemiologists with limited dedicated funds for outbreak response management such as contact tracing. Nigeria, Senegal and Uganda all reported having dedicated budgets for outbreak response which were available at the beginning of the outbreak for provision of personal protective equipment and limited contract tracing [35].

The existing surveillance systems in all the countries were built to respond to localised epidemics whereby the central level response team would support the decentralized rapid response team (RRT) at the outset of an outbreak. However, the subnational structures have never been activated into full preparedness response mode. At the same time the countries were never prepared for simultaneous country-wide outbreak response in multiple geographic locations. In the event of a widespread pandemic such as COVID-19, all countries experienced challenges in subnational responses including shortages in adequately trained surveillance officers, data analyst and contact tracers. Building the capacity of subnational epidemic response capacity requires substantial resources allocation including funding, training staff, and equipping the decentralized centers. Due to resource constraint, a strategy adopted by the countries was to prioritize the districts or regions at highest risk i.e., geographies with higher number of cases. For example, in the DRC, surveillance efforts were strongest in Kinshasa which was the epicentre of the epidemic and where mass testing centres and several diagnostics laboratories were established.

There was deliberate modification in the daily schedule of human resources in communities and at health facilities to reduce their work load and lessen the risk of contracting COVID-19 [12]. Furthermore, countries did not have the appropriate number of responders. For example, there was a lack of adequate contact tracers to match the pandemic demand across all countries. In addition, the study countries still have challenges with ensuring the availability of adequate and appropriately skilled human resource, a situation that preceded the pandemic and that will require strategic resourcing during and after the COVID-19 pandemic. The countries could rapidly train, repurpose, and deploy community-based voluntary health workers (VHW) and facility-based health workers, but these structures require additional strengthening for epidemic preparedness and rapid response.

Overall, analysis and use of surveillance data for action existed at the central/national level but was limited at the subnational levels in all the studied countries. Ensuring timely data availability and use of data is critical to public health decision making. During the pandemic crisis we observed the importance of having the right data which was routinely used for EOC guidance. Therefore, countries had strategies to report data daily, weekly and monthly. The study countries in various degrees adopted electronic systems from paper-based to improve efficiency of data transmission and data use for decision making. Availability of surveillance data contributed to regular review and update of the existing surveillance policies, strategies and standard operating procedures. However, there were notable gaps in data accuracy and consistency at non-sentinel sites than at sentinel sites. Furthermore, the data were not disaggregated by socio-demographic characteristics such as sex, location/place of residence to show burden at the individual level characteristics. The establishment of databases linking surveillance with testing that were accessible to key stakeholders improved communication and efficiency. Data systems development and use require improvement at the subnational levels for more efficient response.

Technological innovations were deployed to enhance surveillance activities including contact tracing, monitoring persons in quarantine, reporting, data analysis, laboratory results return, with improved efficiency. For example, Senegal adopted and used digital communication innovations such as the “Alerte Santé Sénégal” app and “Sunucity” which is an incident reporting app for suspected COVID-19 cases from community that allows feedback from the authorities [12]. These need to be evaluated and replicated.

Strengthening partnerships is key for epidemic preparedness and response. The studied countries were able to source initial supplies with support from non-governmemtal organizations, philanthropists and international partners. The private sector provided funding for testing kits and built facilities for quarantine and isolation services as a surveillance measure. However, the countries need to optimize the public private partnerships in several areas including provision of health services and manufacture of health products. South-to-South COVID-19 response collaboration and technical support can be fostered as a result of the countries’ differential experience. For example, Nigeria and Uganda with expertise in capacity building for epidemic preparedness among responders can be a resource in this area in the region.

Furthermore, there is a need for the improvement of the alert management systems for identification of COVID-19 cases or any other epidemic disease from the community. Senegal, Nigeria and Uganda, reported using call centers with toll free lines to support case identi.fication and contact tracing. However, these call centers were eventually overwhelmed and response became suboptimal as the pandemic progressed with widespread community transmission. Routine alerts could be supplemented with other active surveillance approaches such as systematic health facility surveillance, mortality surveillance and periodic surveys for better estimation of the burden of disease and disease outcomes.

### Study limitations

A substantial part of this study relied on document review. At times the codified evidence in guidelines, policy documents and scientific publication may differ from the real world experience or may lag behind on what could be happening on the ground. Thus, the extent to which the documents reviewed reflected the true practice is uncertain. However, the study mitigated this potential bias by triangulation the literature review information with the qualitative interviews.

This study did not assess the relative performance or effectiveness of surveillance methods. Surveillance methods adopted in the countries studied were complementary. In the face of resource challenges, there is a need to adopt the most comprehensive and cost-effective methods. For example, the European Centre for Disease Preventiion and Control recommended for member countries no longer testing mild suspected cases of COVID-19 should integrate COVID-19 surveillance with sentinel surveillance of influenza-like illness or acute respiratory infection [40]. The swabs obtained at the sentinel sites would then be tested for SARS-CoV-2 in addition to influenza virus. This provides some cost saving by using existing resource framework.

The article showed that private sector contributed immensely to COVID-19 response such as surveillance and testing, treatment, risk communication, health promotion and maintenance of access to essential health services.

## Conclusion

Following the COVID-19 outbreak on the African continent in February- March 2020, the DRC, Nigeria, Senegal and Uganda demonstrated a prompt public health response to the epidemic ass well as instituted national policies aligned to WHO guidance and modified these strategies along the phases of the local epidemic. The countries adopted similar approaches to surveillance, although at different levels and with slight modifications. All four stiudy countries seemingly performed well at the initial stages of preventing transmission through quarantine and isolation. However, as the number of COVID-19 cases began to increase the quarantine and isolation approach began to fault.Coordination of the COVID-19 response in all four study countries built on existing surveillance systems with establishment of central task teams. All countries noted capacity gaps for response at subnational level and adopted electronic systems for data management at varying levels; and they utilized web-based platforms for public data access and visualization. Across four countries, one common challenge was lack of human resource capacity for conducting contact tracing, data analysis, as well as public health expertise.

Recommended approaches such as (1) adopting more innovative technological solutions to improve efficiencies of their surveillance strategies, (2) having a central database across the response pillars to make surveillance more efficient and improve data use at the subnational level, can help stem the further spread of COVID-19, while enhancing readiness for future disease outbreaks. Other approaches include, (3) improvement of human resource surge capacity at subnational level, (4) decentralization of isolation centers and (5) enhancement of home/self-isolation with support from community structures. The private sector should be involved to support response activities, while ensuring proper regulation and quality assurance. Efforts to stem further spread of COVID-19 are critical including roll-out of COVID-19 vaccines, and implementation of targeted non-pharmaceutical interventions.

## Electronic supplementary material

Below is the link to the electronic supplementary material.

Supplementary Material 1

## Data Availability

The datasets used and/or analysed during the current study are available from the corresponding author on reasonable request.
