# Supplementary material for: COVID-19 surveillance in Democratic Republic of Congo, Nigeria, Senegal and Uganda: strengths, weaknesses and key Lessons
Source: BMC Public Health. 2023 May 8;23:835. doi: 10.1186/s12889-023-15708-6 (PMC10165588; doi:10.1186/s12889-023-15708-6)
Supplement: Supplementary file 1 — Supplementary Material 1 [file 12889_2023_15708_MOESM1_ESM.docx]

**Supplementary Materials**

**Additional file 1: Data extraction template**

- File name: Additional file 1
- File format: .doc
- Title of file: Literature abstraction form
- Description of file: contains items for data extraction on strategies, evoluation and challenges of COVID-19 surveillance in four African countries

**Additional file 2: Key informant interview guide**

- File name: Additional file 2
- File format: .doc
- Title of file: Key informant interview guide
- Description of file: Key Informant Guide for Assessment of Covid19 response in Eastern, Central and Western Africa

**Additional file 1: Literature Abstraction Form**

**Detection, Testing and Surveillance:**

**Section A: Detection &Testing**

| 1. **Testing Modalities** | | | | | |
| --- | --- | --- | --- | --- | --- |
|  | What tests have been used (antigen/antibody)?  **What tests have been used (antigen/antibody/molecular test?**  **"What testing strategies(high risk or mass , corporate testing, community testing, post mortem testing)**  **• Who should be tested? (eligibility criteria)**  **• When and with what frequency?**  **• How should they be tested? (using what testing modalities)**  **To make what decisions (Isolation, quarantine, discharge etc ) "**  **How has the strategy changed over time and why** | | | | |
|  | What testing strategies (high risk or mass, corporate testing, community testing, post mortem testing)?  **How was the country able to increase testing capacity? How quickly was testing ramped up? Document strategies employed to increase testing capacity?**  **Document the key strengths and weaknesses of each strategy.**  **Document what precipitated + enabled major shifts in testing numbers?**  **Document how any weaknesses were addressed or mitigated.**  **"Test quality:**  **Document the % of samples tested accurately.**  **How is Quality Assurance (QA) monitored and ensured?**  **"**  **Document the Turnaround time (TAT) from sample collection to results return?**  **"Document strategies in place to improve testing TAT and ensure timely return of results to**  **Tested population;**  **Health providers;**  **Public Health decision makers;**  **"**  **Describe the national IT infrastructure/existing data systems for public health decision makers and how this has been modified following the COVID-19 pandemic** | | | | |
|  | How has testing changed over the past 8 months? **Yes=1; No=2 (please circle)**  If either Yes or No, Why?  **"What is the # of covid testing sites set up?**  **Distribution of lab testing sites across the country?**  **What guided site distribution?**  **Type of sites used (e.g., new COVID capacity, HIV testing)**  **"**  **"Document the testing capacity of each lab or the national daily testing capacity.**  **Testing capacity per site (number of samples /day)?**  **How did this change over time?**  **"**  **What % of health facilities have access to test kits (should approach 100%)**  **Document the median TAT from sample collection to delivery to the testing site /lab**  **"What % samples arrive in good condition?**  **Document the sample rejection rates (and criteria for sample rejection)**  **"**  **Describe the sample referral system and strategies to improve TAT**  **Document key strengths, weaknesses, challenges, gaps in sample delivery to lab from collection**  **Lab testing key strengths and chellenges**  **Cumulative tests per capita as per 31st october 2020 (Key strangths weaknesses challenges and gaps)**  **Cumulative tests per confirmed case (Key strangths weaknesses challenges and gaps)**  **Document trends in the ‘test per case ratio’ i.e. # of tests performed per +ve case. How has this changed over time? (Key strangths weaknesses challenges and gaps)**  **Cumulative tests per confirmed death as per 31st October 2020 (Key strangths weaknesses challenges and gaps)**  **Repeat tests as per 31st october 2020** | | | | |
|  | Cumulative tests per capita as per 31st October 2020 | | |  | |
|  | Cumulative tests per confirmed case | | |  | |
|  | Cumulative tests per confirmed death as per 31st October 2020 | | |  | |
|  | Repeat tests as per 31st October 2020 | | |  | |
|  | When were the country COVID-19 testing policies, strategies, models and algorithms finalized and disseminated/communicated to stakeholders?  **When were the country COVID-19 testing policies, strategies, models and algorithms finalized and disseminated/ communicated to stakeholders ? (Key strangths weaknesses challenges and gaps)** | | | | |
|  | 1. testing policies | |  | | |
|  | 1. testing strategies | |  | | |
|  | 1. testing models | |  | | |
|  | 1. testing algorithms | |  | | |
| 1. **Changes in testing criteria** | | | | | |
|  | How has testing changed over the past 8 months?  **How has testing criteria changed over the past 8 months?** | | | | |
|  | Why?  **Why were these changes there?**  **How has access to testing been modified over time?**  **Document any groups that may have challenges in accessing testing?**  **"Document factors affecting demand for testing in relation to country capacity and how this is managed by the country**  **Excessive demand**  **Inadequate demand**  **"**  **What are the key barriers to testing? What strategies are in place to increase demand for testing?**  **When over/underutilized on capacity, what messaging /communications were used, etc.**  **Document key strengths, weaknesses, challenges, gaps in sample collection and access to testing** | | | | |
| 1. **Document the source of the COVID-19 test kits and supplies** | | | | | |
|  | Are test kits locally manufactured / Imported / Donated? (Start date ) | | | | |
|  | - locally manufactured | Yes=1; No=2 (please circle) | | | Start Date: |
|  | - Imported | Yes=1; No=2 (please circle) | | | Start Date: |
|  | - Donated? | Yes=1; No=2 (please circle) | | | Start Date: |
|  | What is the proportion / number of test kits produced domestically or imported? | | |  | |
|  | What is the manufacturing capacity? | | |  | |
|  | Time to produce new kits | | |  | |

**"What method was used to deliver testkits and supplies to testing centers?**

**Document the median time to receive kits after an order is placed**

**"**

**Document any stock outs experienced, when and why?**

**Document the strengths and weaknesses / gaps observed in sourcing and distribution of test kits?**

**"Are the tests provided at testing centers free of charge/ are they paid for?**

**Document the price of the test to the population? Trends in pricing …**

**Who funds the testing? " (Key strangths weaknesses challenges and gaps )**

**Section B: Surveillance**

| 1. **Modalities of implementing contact tracing** | | | | |
| --- | --- | --- | --- | --- |
|  | Duration/level of implementation:  **What happened to those with positive screen test**  **(Key strangths weaknesses challenges and gaps)** | | | |
|  | Who was traced? |  | | |
|  | By whom? |  | | |
|  | Changes in policies on contact tracing **(Key strangths weaknesses challenges and gaps)** |  | | |
| 1. **Modalities of implementing isolation** | | | | |
|  | Duration/level of implementation (e.g. centralized isolation, home isolation):  **Document country policy on treatment and quarantine**  **What treatment or quarantine options exist?**  **What is the isolation and quarantine strategy – who, where, and for how long**  **Document the quarantine capacity compared to demand? Are sites equitably distributed?**  **"Document strategies to facilitate:**  **Regular monitoring of quarantine subjects**  **How is adherence to quarantine tracked?**  **"**  **How is the home-isolation monitored and supported?**  **"Document Key strengths, challenges, gaps in isolation and qurantine implementation. Document strategies to address the gaps and challenges**  **" (Key strangths weaknesses challenges and gaps)**  **"Changes in policies on isolation and quarantine.**  **How has the strategy changed over time"**  **What influenced the change in policy/ strategy ?**  **(Key strangths weaknesses challenges and gaps)** | | | |
|  | Changes in policies on contact tracing |  | | |
| 1. **Modalities of screening (temperature screening)** | | | | |
|  | 1. How it was implemented? | |  | |
|  | 1. Where? | |  | |
|  | 1. Who was screened? | |  | |
|  | 1. What happened to those with positive screen test? | |  | |
|  | 1. Changes in temperature screening? | |  | |
|  | **(Key strangths weaknesses challenges and gaps)** | |  | |
|  | **Changes in policies on screening** | |  | |
| 1. **Surveillance Data** | | | | |
|  | 1. Data completeness (reporting rates) | | |  |
|  | 1. Cases per capita as per 31st October 2020 | | |  |
|  | 1. Cases doubling time | | |  |
|  | 1. Deaths per capita, as per 31st October 2020 | | |  |
|  | 1. Deaths doubling time | | |  |
|  | 1. Changes in isolation criteria | | |  |
|  | 1. Changes in quarantine criteria | | |  |
|  | 1. (i) Were any surveys done? | | |  |
|  | 1. How did the data from the surveys inform response? | | |  |
|  | 1. **(Key strangths weaknesses challenges and gaps)** | | |  |

***Note: Please feel free to expand the cells if more information is required***

**Describe the Early Warning, Alert & Response System.**

**Who was screened by the EWARN system?**

**Document procedures to improve case detection in the community**

**Desribe procedures to improve reporting of suspect cases**

**Modalities to improve referral of suspect cases**

**(Key strangths weaknesses challenges and gaps)**

**What % of COVID-19 positive patients recieved results?**

**What % of COVID-19 positive patients were isolated?**

**What % of COVID-19 positive patients had contacts traced?**

**(Key strangths weaknesses challenges and gaps)**

**Definition:Turn Around Time (TAT) is the total time taken between the submission of a program/process/task for execution and the return of the complete output to the customer/user**

**Document the median TAT from sample collection to delivery to the testing site /lab.**

**What were the sample rejection rates**

**What were the criteria for sample rejection?**

**(Key strangths weaknesses challenges and gaps)**

**How has existing surveillance system been affected by COVID 19?**

**What additional surveillance capacity was instituted due to covid-19 pandemic**

**(Key strangths weaknesses challenges and gaps)**

**Thank you.**

**Additional file 2: Key Informant Interview Guide**

**Key Informant Guide for Assessment of Covid19 response in Eastern, Central and Western Africa**

**Key informant Interview Guide- Policy Maker (**Head Emergency Operation Centre, Head Coordination pillar, Head Case management pillar, Head Epidemiology and Surveillance Department, Ministry of Health)

**Title:** COVID-19: Assessment of the COVID-19 Response in Eastern, Central and Western Africa

**Identifiers**

1. Institution:
2. Office/Position:
3. Duration in the position:
4. Age in complete years:
5. Gender:
6. Profession:
7. Role in COVID-19 response:
8. Can you describe the national administrative structure and whether the response to COVID-19 varied by administrative/ federal structure/composition?

**Section A Past Experience with Epidemics**

1. How have experiences with previous epidemics been useful in the COVID-19 response (experiences in coordination, surveillance, testing etc.)
2. How did the country respond? (Probe on successes, challenges/gaps, lessons, recommendations)?

**Section B: Health System preparedness (specifically about funding)**

1. How were the resources (funds/financing) to respond to COVID19 obtained? (Country budget allocations, domestic/international funding, what it supported (kind/cash))

**Public health infrastructure**

1. Countries have based on existing systems to respond to covid-19. What modifications were made to ensure effective response for covid-19 and why? i.e What systems and structures existed for COVID-19 response? How were these structures/systems used during COVID 19 response?, How did these mechanism/systems evolve during COVID 19 response. (*Probe deeper depending on the respondent: what existed, what was new, what worked centrally/ provincially/regionally or at the district, and how it worked?)*
   1. Coordination of responses
   2. Surveillance? (*inquire if countries did surveys, post mortem surveillance, workplace surveys and surveillance, etc)*
   3. Epidemic information systems?
   4. Contact tracing?
   5. Laboratory infrastructure
   6. Risk Communication/ Social Mobilization
   7. Logistics and commodities
   8. Human resources (What mechanisms existed for building capacity for human resources?
   9. Isolation?
   10. Quarantine (Institutional to home-based)?
   11. Treatment (Hospital capacity to manage epidemics, ICU Capacity, human resources, bed capacity, PPE, laboratory capacity, medicines, equipment, how hospital capacity was strengthened to handle COVID19 challenges (Probe for designated facilities? Increased human resources? Increased protective gear

1. Has there been any rapid community surveys to assess prevalence of COVID-19 in the community (Probe for survey dates and findings)
2. What strategies were used for testing? How effective was the testing strategy? How did these change during COVID 19 response and why? How did the tests per capita improve? Does the country have the capacity to produce testing kits locally? What is the sustainability plan for Covid19 testing in the country? In light with the current COVID19 pandemic, what do you think of the 1^st^ wave and the 2^nd^ wave? What testing and surveillance strategies have you put in place and have they been changed over the different phases of the pandemic
3. Where there any displacements of services where testing for Covid19 used GeneXpert, or PCR testing? To what extent was the displacement of diagnostics? How has this affected service delivery and what options are available to address these diagnostic displacements?
4. With new COVID19 variants (mutations) coming up, how is the country preparing to cope and respond? (Is there surveillance strategy for looking out for the new variants? Any ongoing genotypic / sequencing tests in the country?
5. COVID-19 vaccination (Probe about vaccine preparedness, plans, funding, key challenges/barriers). (what plans are in place currently, what is the agreed criteria for vaccine prioritization, who is funding vaccinations, vaccine logistics preparations, what challenges do you anticipate?)

**Section C: Response to the COVID-19 pandemic in Africa and the outcomes in terms of Nonpharmaceutical COVID-19 control strategies**

1. What NPI strategies were implemented in the country?
   1. Lock down (probe for exact dates when they were instituted, geographical coverage, for how long and why?)
   2. Social distance (probe for exact dates when they were instituted, geographical coverage, for how long and why?)
   3. Sanitation/Hygiene (probe for exact dates when they were instituted, for how long and why?)
   4. Masks (probe for exact dates when they were instituted, for how long and why?)
   5. Quarantine measures (probe for exact dates when they were instituted, geographical coverage, for how long and why?)
   6. Isolation (probe for exact dates when they were instituted, for how long and why?)
   7. Curfew (probe for exact dates when they were instituted, for how long and why?)
2. In your opinion, how effective were each of the interventions in limiting the spread of COVID-19 and why? [Extremely effective, very effective, moderately effective, slightly effective or not effective at all?]
3. Lockdown
4. Social distancing
5. Masks
6. Sanitation and Hygiene
7. Quarantine measures
8. Isolation
9. Curfew
10. In your opinion to what extent did the public comply with each of the interventions to limit spread of COVID-19 and why? [Extremely compliant, very compliant, moderately compliant, slightly compliant or not compliant at all?]
11. Lockdown
12. Social distancing
13. Masks
14. Sanitation and Hygiene
15. Quarantine measures
16. Isolation
17. Curfew

**Section D: Strategies to ensure Continuity of essential non-COVID services**

1. What strategies were used to ensure continuity of care for different essential services like
2. Maternal and child health
3. Immunization
4. Family Planning Services
5. Non-Communicable Diseases like diabetes and high blood pressure
6. HIV/TB Services
7. Paediatric OPD attendance
8. Other country specific services
9. How effective were these strategies? [Extremely effective, very effective, moderately effective, slightly effective or not effective at all?]
10. What challenges were faced in the maintenance of these services?

**Section E: Positive or negative unintended consequences including socioeconomic and gender related vulnerability**.

1. What were the unintended consequences of the interventions to control COVID-19? (Ask for positive and negative) (Probes: gender-based violence, teenage pregnancy, education, poverty, food insecurity, economic opportunities, unemployment rates, child labour, out of pocket expenditure for health, mental disorders like depression, suicides, per capita income, health spending as percentage of GDP, health insurance, inflation rate)To what extent did COVID-19 and NPIs to control it result into socio-economic vulnerability? How was the impact of COVID-19 impact different for men, women, children? How about for the socially vulnerable communities including those living in slums, the rural poor, refugees, the homeless, migrant workers, those in the informal sector, persons with disabilities
2. Which COVID-19 interventions had the most impact on gender and socio-economic vulnerability negatively or positively?

What strategies did government use to minimize socio economic consequences of COVID 19? To whom were the measures targeted? How were they implemented?

1. How effective were these strategies? [Extremely effective, very effective, moderately effective, slightly effective or not effective at all?] What mechanisms did individuals themselves adopt to cope with the gender and socio-economic impacts of COVID-19 restrictions?
2. To what extent did COVID 19 result in gender-based violence escalation in your country?
3. What strategies did government use to minimize gender-based violence consequences of COVID-19?
4. How effective were these strategies? [Extremely effective, very effective, moderately effective, slightly effective or not effective at all?]

#

# Appendix V: Key informant Interview Guide_ Epidemic Focal Person (Surveillance focal person, Regional surveillance focal person, Incident commander, Pillar head Surveillance and lab, Contact tracing head, risk communication lead, Alert desk lead, IPC head (Isolation and quarantine), Cross-border surveillance officers)

**Title:** COVID-19: Assessment of the COVID-19 Response in Eastern, Central and Western Africa

**Identifiers**

1. Institution:
2. Office/Position:
3. Duration in the position:
4. Age in complete years:
5. Gender:
6. Profession:
7. Role in COVID-19 response:
8. Can you describe the national administrative structure and whether the response to COVID-19 varied by administrative/ federal structure?

**Section A Past Experience with Epidemics**

1. How has the experiences with previous epidemics been useful in the COVID-19 response (experiences in coordination, surveillance, testing etc)
2. How did the country respond? (Probe on successes, challenges/gaps, lessons, recommendations)?

**Section B: Health System preparedness (specifically about funding)**

1. How were the resources (funds/financing) to respond to COVID1-9 obtained ()? (Country budget allocations, domestic/international funding, what it supported (kind/cash))

**Public health infrastructure**

1. Countries have based on existing systems to respond to covid-19. What modifications were made to ensure effective response for covid-19 and why? i.e What systems and structures existed for COVID-19 response? How were these structures/systems used during COVID 19 response?, How did these mechanism/systems evolve during COVID 19 response. (*Probe deeper depending on the respondent: what existed, what was new, what worked centrally/ provincially/regionally or at the district, and how it worked?)*
   1. Coordination of responses
   2. Surveillance? (*inquire if countries did surveys, post mortem surveillance, workplace surveys and surveillance, etc)*
   3. Epidemic information systems?
   4. Contact tracing?
   5. Laboratory infrastructure
   6. Risk Communication/ Social Mobilization
   7. Logistics and commodities
   8. Human resources (What mechanisms existed for building capacity for human resources?
   9. Isolation
   10. Quarantine
   11. Treatment (Hospital capacity to manage epidemics, ICU Capacity, human resources, bed capacity, PPE, laboratory capacity, medicines, equipment, how hospital capacity was strengthened to handle COVID challenges (Probe for designated facilities? Increased human resources? Increased protective gear

1. What strategies were used for testing? How effective was the testing strategy? How did these change during COVID 19 response and why? How did the tests per capita improve? Does the country have the capacity to produce testing kits locally? What is the sustainability plan for Covid19 testing in the country? In light with the current COVID19 pandemic, what do you think of the 1^st^ wave and the 2^nd^ wave? What testing and surveillance strategies have you put in place and have they been changed over the different phases of the pandemic
2. Where there any displacements of services where testing for Covid19 used GeneXpert, or PCR testing? To what extent was the displacement of diagnostics? How has this affected service delivery and what options are available to address these diagnostic displacements?
3. With new COVID19 variants (mutations) coming up, how is the country preparing to cope and respond? (Is there surveillance strategy for looking out for the new variants? Any ongoing genotypic / sequencing tests in the country?
4. COVID-19 vaccination (Probe about vaccine preparedness, plans, funding, key challenges/barriers). (what plans are in place currently, what is the agreed criteria for vaccine prioritization, who is funding vaccinations, vaccine logistics preparations, what challenges do you anticipate?)

**Section C: Response to the COVID-19 pandemic in Africa and the outcomes in terms of Nonpharmaceutical COVID-19 control strategies (Provide evidence of guidelines, SOPs, reports etc)**

1. What NPI strategies were implemented in the country?
   1. Lock down (probe for exact dates when they were instituted, geographical coverage, for how long and why?)
   2. Social distance (probe for exact dates when they were instituted, geographical coverage, for how long and why?)
   3. Sanitation/Hygiene (probe for exact dates when they were instituted, for how long and why?)
   4. Masks (probe for exact dates when they were instituted, for how long and why?)
   5. Quarantine measures (probe for exact dates when they were instituted, geographical coverage, for how long and why?)
   6. Isolation (probe for exact dates when they were instituted, for how long and why?)
   7. Curfew (probe for exact dates when they were instituted, for how long and why?)
2. In your opinion, how effective were each of the interventions in limiting the spread of COVID-19? [Extremely effective, very effective, moderately effective, slightly effective or not effective at all?]
3. Lockdown
4. Social distancing
5. Masks
6. Sanitation and Hygiene
7. Quarantine measures
8. Isolation
9. Curfew
10. In your opinion to what extent did the public comply with each of the interventions to limit spread of COVID-19? [Extremely compliant, very compliant, moderately compliant, slightly compliant or not compliant at all?]
11. Lockdown
12. Social distancing
13. Masks
14. Sanitation and Hygiene
15. Quarantine measures
16. Isolation
17. Curfew

**Section D: Strategies to ensure Continuity of essential non-COVID services**

1. What strategies were used to ensure continuity of care for different essential services like
2. Maternal and child health
3. Immunization
4. Family Planning Services
5. Non Communicable Diseases like diabetes and high blood pressure
6. HIV/TB Services
7. Paediatric OPD attendance
8. Other country specific services
9. How effective were these strategies? [Extremely effective, very effective, moderately effective, slightly effective or not effective at all?]
10. What challenges were faced in the maintenance of these services?

**Section E: Positive or negative unintended consequences including socioeconomic and gender related vulnerability**.

1. What were the unintended consequences of the interventions to control COVID-19? (Ask for positive and negative) (Probes: gender-based violence, teenage pregnancy, education, poverty, food insecurity, economic opportunities, unemployment rates, child labour, out of pocket expenditure for health, mental disorders like depression, suicides, per capita income, health spending as percentage of GDP, health insurance, inflation rate)To what extent did COVID-19 and NPIs to control it result into socio-economic vulnerability? How was the impact of COVID-19 impact different for men, women, children? How about for the socially vulnerable communities including those living in slums, the rural poor, refugees, the homeless, migrant workers, those in the informal sector, persons with disabilities
2. Which COVID-19 interventions had the most impact on gender and socio-economic vulnerability negatively or positively?

What strategies did government use to minimize socio economic consequences of COVID 19? To whom were the measures targeted? How were they implemented?

1. How effective were these strategies? [Extremely effective, very effective, moderately effective, slightly effective or not effective at all?] What mechanisms did individuals themselves adopt to cope with the gender and socio-economic impacts of COVID-19 restrictions?
2. To what extent did COVID 19 result in gender-based violence escalation in your country?
3. What strategies did government use to minimize gender-based violence consequences of COVID-19?
4. How effective were these strategies? [Extremely effective, very effective, moderately effective, slightly effective or not effective at all?]

**Appendix VI: Key informant Interview _Health manager (COVID-19 and general facilities)** (Quarantine units, Isolation units, Treatment units, Lab manager (private/public), Public-private Partnerships (test kits and investments required), Psychosocial/community engagements, DHO (preparedness and capacity building efforts) (border and non-border/Capital City Authority)

**Title:** COVID-19: Assessment of the COVID-19 Response in Eastern, Central and Western Africa

**Identifiers**

1. Institution:
2. Office/Position:
3. Duration in the position:
4. Age in complete years:
5. Gender:
6. Profession:
7. Role in the covid-19 response:

**Section B: Facility preparedness and Public health infrastructure**

1. What systems or mechanisms were used [**before** the COVID-19 pandemic]
   1. Screening?
   2. Testing?
   3. Reporting?
   4. Managing patients?
   5. Managing contacts?
   6. Contact tracing?
   7. Logistics and commodities
   8. Isolation
   9. Quarantine
2. How were these mechanism/systems used **during** COVID 19 response?
3. How did these change during COVID 19 response?
   1. Screening?
   2. Testing?
   3. Reporting?
   4. Managing patients?
   5. Managing contacts?
   6. Contact tracing?
   7. Logistics and commodities
   8. Isolation
   9. Quarantine
4. What was hospital capacity to manage epidemics in terms of(specify if capacity was strengthened during the response)
   1. Laboratory capacity
   2. Human resources
   3. Infection Prevention and Control
   4. Personal Protective Equipment (PPE) for health workers
   5. General bed Capacity
   6. ICU Bed capacity
5. How was hospital capacity strengthened to handle COVID-19 challenges (probe for designated facilities?
6. How was testing/screening/laboratory capacity increased during the response?
7. How were human resources increased?
8. What were the interventions to increased protective gear?
9. Test kits and investments required (For Public-Private Partnerships Managers)
10. Resource (funds) availability
11. Data flow process
12. What were the implementation challenges/gaps?
13. What were the recommendations/innovations?

**Section C: Response to the COVID-19 pandemic in Africa and the outcomes in terms of Non-pharmaceutical COVID-19 control strategies**

1. Contact tracing (probe for exact dates how it was done, for how long and why?)
2. Isolation (probe for exact dates when they were instituted, geographical coverage, for how long and why?)
3. Curfew: (probe for exact dates when they were instituted, geographical coverage, for how long and why?)
4. How effective were these strategies? [Extremely effective, very effective, moderately effective, slightly effective or not effective at all?]

**Section D: Strategies to ensure Continuity of essential non-COVID**

1. What strategies were used to ensure continuity of care for different essential services like
   1. Maternal and child health
   2. Immunization
   3. Family Planning Services
   4. Non-Communicable Diseases like diabetes and high blood pressure
   5. HIV/TB Services
   6. Pediatric OPD attendance
   7. Other country specific services
2. How effective were these strategies? [Extremely effective, very effective, moderately effective, slightly effective or not effective at all?]

**Section E: Positive or negative unintended consequences including socioeconomic and gender related vulnerability**.

1. What were the unintended consequences of the interventions to control COVID-19? (Ask for positive and negative) (Probes: gender-based violence, teenage pregnancy, education, poverty, food insecurity, economic opportunities, unemployment rates, child labour, out of pocket expenditure for health, mental disorders like depression, suicides, per capita income, health spending as percentage of GDP, health insurance, inflation rate)To what extent did COVID-19 and NPIs to control it result into socio-economic vulnerability? How was the impact of COVID-19 impact different for men, women, children? How about for the socially vulnerable communities including those living in slums, the rural poor, refugees, the homeless, migrant workers, those in the informal sector, persons with disabilities
2. Which COVID-19 interventions had the most impact on gender and socio-economic vulnerability negatively or positively?

What strategies did government use to minimize socio economic consequences of COVID 19? To whom were the measures targeted? How were they implemented?

1. How effective were these strategies? [Extremely effective, very effective, moderately effective, slightly effective or not effective at all?] What mechanisms did individuals themselves adopt to cope with the gender and socio-economic impacts of COVID-19 restrictions?
2. To what extent did COVID 19 result in gender-based violence escalation in your country?
3. What strategies did government use to minimize gender-based violence consequences of COVID-19?
4. How effective were these strategies? [Extremely effective, very effective, moderately effective, slightly effective or not effective at all?]
